# Radiation Oncology reviewer acknowledgment 2012

**DOI:** 10.1186/1748-717X-8-63

**Published:** 2013-04-05

**Authors:** Claus Belka

**Affiliations:** 1 Department of Radiotherapy and Radiation Oncology, Ludwig-Maximilians, University Munich, Marchioninistr 15, 81377 Munich, Germany

## Abstract

**Contributing reviewers:**

The editor of *Radiation Oncology* would like to thank all our reviewers who have contributed to the journal in Volume 7 (2012).

## 

Lauren Abrey

Switzerland

Mario Airoldi

Italy

Ichiro Akagi

Japan

David Allan

Canada

Filippo Alongi

Italy

Michelle Alonso-Basanta

United States of America

Dante Amelio

Italy

Richard Amos

United States of America

Franc Anderluh

Slovenia

Fundagul Andic

Turkey

Nicolaus Andratschke

Germany

Carmen Ares

Switzerland

Cynthia Aristei

Italy

Villers Arnauld

France

Jonathan Ashman

United States of America

Vasileios Askoxylakis

Germany

Avi Assouline

France

David Azria

France

Koichi Azuma

Japan

Matthias Bache

Germany

Kate Bak

Canada

David Ball

Australia

Voichita Bar Ad

United States of America

Isabelle Barillot

France

Thomas Barr

United States of America

Bahadir Batar

Turkey

Glenn Bauman

Canada

Martina Baumann

Austria

Brigitta G. Baumert

Netherlands

Siddhartha Baxi

Australia

Beth Beadle

United States of America

Christoph Beier

Germany

Jose Belderbos

Netherlands

Claus Belka

Germany

Susan Bellis

United States of America

Stanley Benedict

United States of America

Bernhard Berger

Germany

Anna Sophie Berghoff

Austria

Christoph Bert

Germany

Anders Bertelsen

Denmark

Nuran Senel Bese

Turkey

Shreerang Bhide

United Kingdom

Jean-Emmanuel Bibault

France

Marc Bischof

Germany

Giampaolo Biti

Italy

Jon Kroll Bjerregaard

Denmark

Judit Boda-Heggemann

Germany

Liesbeth Boersma

Netherlands

Carl Bogardus

United States of America

Vikram Rao Bollineni

Netherlands

Paul Bonnitcha

Australia

Alberto Bossi

France

Dirk Bottke

Germany

Christian Boy

Germany

Georg Brabant

United Kingdom

Julie Bradley

United States of America

Gregor Bran

Germany

Sara Broggi

Italy

Thomas Brunner

United Kingdom

John Buatti

United States of America

Jeroen Buijsen

Netherlands

Guillaume Buiret

France

Murat Caloglu

Turkey

Kevin Camphausen

United States of America

Carme Camps

United Kingdom

Dexter Canoy

United Kingdom

Francesco Caponigro

Italy

Dennis Carter

United States of America

Antony Carver

United Kingdom

Jason Cashmore

United Kingdom

Douglas Castro

Brazil

Jean-Michel Caudrelier

Canada

Laura Cella

Italy

Giovanni Luca Ceresoli

Italy

Arnab Chakravarti

United States of America

Annie Chan

United States of America

Zheng Chang

United States of America

Daniel Chang

United States of America

Samuel Chao

United States of America

Ming Chun Chau

Hong Kong

Ronald Chen

United States of America

Yi-Jen Chen

United States of America

Wei Chen

United States of America

Changhu Chen

United States of America

Harry Cheng

Hong Kong

Jason Chia-Hsien Cheng

Taiwan

James Chow

Canada

Judith Christian

United Kingdom

Carrie Christian

French Guiana

David Christiani

United States of America

Daniel Chua

Hong Kong

Savino Cilla

Italy

Catharine Clark

United Kingdom

Brenda Clark

Canada

Alessandro Clivio

Switzerland

Gil Cohen

United States of America

Federico Collettini

Germany

Stephanie E. Combs

Germany

Robin Cornelissen

Netherlands

Renzo Corvo

Italy

Scott Cozad

United States of America

Luca Cozzi

Switzerland

Gilles Crehange

France

Juliane Daartz

United States of America

Kirsten Dahm

Germany

Savita Dandapani

United States of America

Prajnan Das

United States of America

Carol-Anne Davis

Canada

Regina Day

United States of America

Berardino De Bari

France

Gert De Meerleer

Belgium

Letizia Deantonio

Italy

Daviod Dearnaley

United Kingdom

Pradip Deb

Australia

Annelies Debucquoy

Belgium

Thomas Delaney

United States of America

Carmen Dence

United States of America

Kristopher Dennis

Canada

Beatrice Detti

Italy

Curtiland Deville

United States of America

Robert Diaz Beveridge

Spain

Adam Dicker

United States of America

Karin Dieperink

Denmark

Tim Dijkema

Netherlands

Maktav Dincer

Turkey

George Ding

United States of America

Rob Dinniwell

Canada

Joseph Disa

United States of America

Chaitanya Divgi

United States of America

Ivan Dobri

Croatia

Werner Dobrowsky

United Kingdom

Nesrin Dogan

United States of America

Yuichiro Doki

Japan

Andrew Donson

United States of America

Ilana Doweck

Israel

Anthony Dragun

United States of America

Isabelle Dublineau

France

Francois Ducray

France

Fréderic Duprez

Belgium

Marco Durante

Germany

Vincent Duronio

Canada

Michael Eble

Germany

Anne Edvardsen Kyrdalen

Norway

Ann Marie Egloff

United States of America

Avraham Eisbruch

United States of America

Issam El Naqa

Canada

Helene Elleaume

France

Benedikt Engels

Belgium

Rita Engenhart-Cabillic

Germany

Iris Ernst

Germany

Floris Ernst

Germany

Natia Esiashvili

United States of America

Ehab Esmat

Saudi Arabia

Samer Ezziddin

Germany

Khashayar Fakhrian

Germany

Fu-Min Fang

Taiwan

Daniel Fass

United States of America

Horst Feldmann

Germany

Pascal Fenoglietto

France

Aaron Fenster

Canada

Alfio Ferlito

Italy

Annemarie Fernandes

United States of America

Vladimir Feygelman

United States of America

Isabelle Fitton

France

Markus Fitzek

United States of America

Jochen Fleckenstein

Germany

Antonella Fogliata

Switzerland

Dominic Fong

Italy

Silvia Formenti

United States of America

Piero Fossati

Italy

Giovanni Franchin

Italy

Gijs Franssen

Netherlands

John French

Canada

Anna Friedl

Germany

Mitsuhiro Fujishiro

Japan

Jurgen Futterer

Netherlands

Giovanna Gagliardi

Sweden

Udo Gaipl

Germany

Suthakar Ganapathy

United States of America

Adam Garden

United States of America

Madhur Garg

United States of America

David Geffen

Israel

Dietmar Georg

Austria

Moshi Geso

Australia

David Gewirtz

United States of America

Pirus Ghadjar

Switzerland

David Gierga

United States of America

Marinello Ginette

France

Jordi Giralt

Spain

Andrea Girlando

Italy

Alix Gitelman

United States of America

Gregor Goldner

Austria

Daniel Gomez

United States of America

Youling Gong

China

Karyn Goodman

United States of America

Peter Greer

Australia

Laval Grimard

Canada

Stephen Grobmyer

United States of America

Guenther Gruber

Switzerland

Janneke Grutters

Netherlands

Ferran Guedea

Spain

Chandan Guha

United States of America

Akos Gulyban

Belgium

Junming Guo

China

Tejpal Gupta

India

Angelita Habr-Gama

Brazil

Andreas Hadjisavvas

Cyprus

Janet Hall

France

Ian Noel Hampson

United Kingdom

Chunhui Han

United States of America

Adriana Handra-Luca

France

Alexandra Hanlon

United States of America

Sheng-Po Hao

Taiwan

Hideyuki Harada

Japan

Andrew Hartley

United Kingdom

Masaharu Hata

Japan

Jona Hattangadi

United States of America

Joan Hatton

Australia

Maria Hawkins

United Kingdom

Emily Heath

Canada

Michael Heckman

United States of America

Martinus Heesters

Netherlands

Christophe Hennequin

France

Joseph Herman

United States of America

Reinhard Heyd

Germany

Guido Hildebrandt

Germany

Ying Hitchcock

United States of America

Maritza Hobson

Canada

Wolfgang Hoegele

Germany

Martin Holcik

Canada

Dennis Holmes

United States of America

Ji-Hong Hong

Taiwan

Bradford Hoppe

United States of America

Janet Horton

United States of America

Laurent Houzet

Armenia

Morten Høyer

Denmark

Yu-Jie Huang

Taiwan

Kitty Huang

Canada

Yung-Hui Huang

Taiwan

Michael Hughes

United States of America

Florence Huguet

France

John Humm

United States of America

Coen Hurkmans

Netherlands

Chris Hurt

United Kingdom

Hiroshi Igaki

Japan

Daniel Indelicato

United States of America

Fumiaki Isohashi

Japan

Traivs Jacobson

United States of America

Ganesh Chandra Jagetia

India

Rakesh Jalali

India

Nathalie Jansen

Germany

Verena Jendrossek

Germany

Alexandra D. Jensen

Germany

Branislav Jeremic

Serbia

Bruno Jham

United States of America

Keiichi Jingu

Japan

Bleddyn Jones

United Kingdom

Johannes Kaanders

Netherlands

Matthew Kalady

United States of America

Shalom Kalnicki

United States of America

Maria Kamstrup

Denmark

Tatsuo Kanda

Japan

Ki Mun Kang

Korea, South

Omur Karakoyun Celik

Turkey

Genro Kashino

Japan

Katsuhiko Kashiwa

Japan

Ikuo Kashiwakura

Japan

Alan Katz

United States of America

Harvey Katzen

United States of America

Shinji Kawabata

Japan

Dmitry Kazakov

Czech Republic

Paul Keall

Australia

Chris Kelsey

United States of America

Lucyna Kepka

Poland

Joost Kerklaan

Netherlands

Larry Kestin

United States of America

Elizabeth Kidd

United States of America

Young Tae Kim

Korea, South

Sungheon Kim

United States of America

Dae Yong Kim

Korea, South

Siyong Kim

United States of America

In Ah Kim

Korea, South

Anna Kirby

United Kingdom

Chifumi Kitanaka

Japan

Philip Kleine

Germany

Martin Kocher

Germany

Julia Koeck

Germany

Oliver Koelbl

Germany

Neil Kopek

Denmark

Rolf-Dieter Kortmann

Germany

Gyoergy Kovacs

Germany

Wilma Kraft-Weyrather

Germany

Mechthild Krause

Germany

Sonja Krause

Germany

Leos Kren

Czech Republic

Marco Krengli

Italy

Steven Kronowitz

United States of America

Stephen Kry

United States of America

Hiroyuki Kuwano

Japan

Frank Lagerwaard

Netherlands

Susan Lalondrelle

United Kingdom

Valeria Landoni

Italy

Hans Langendijk

Netherlands

Keith Langmack

United Kingdom

Tanja Langsenlehner

Austria

Constantin Lapa

Germany

Normand Laperriere

Canada

Wolfram Laub

United States of America

Tim Lautenschlaeger

United States of America

Roberta Lazzari

Italy

W. Robert Lee

United States of America

Hsin-Lun Lee

Taiwan

Baosheng Li

China

Minglun Li

Germany

Libo Li

China

Lilie Lin

United States of America

Zhixiong Lin

China

Kant Lin

United States of America

Katja Lindel

Germany

Zhuang Ling

United States of America

Guilin Liu

Australia

Stanley Liu

Canada

Lorenzo Livi

Italy

Fabian Lohaus

Germany

Frank Lohr

Germany

Maria Loizidou

Cyprus

Geneviève LOOS

France

Thomas Losasso

United States of America

Philippe Lothaire

Belgium

Laura Lozza

Italy

Hsiao-Ming Lu

United States of America

Jiade Lu

Singapore

Jiade J. Lu

China

Giovanni Lucignani

Italy

Michelle Ludwig

United States of America

LD Lunsford

United States of America

Stephen Lutz

United States of America

Xuejun Ma

China

Jun Ma

China

Nicola Mabjeesh

Israel

Gabriella Macchia

Italy

Shannon Macdonald

United States of America

Mitchell Machtay

United States of America

William Mackillop

Canada

Indira Madani

Belgium

Philippe Maingon

France

Robert Malyapa

United States of America

Farkhad Manapov

Germany

Pietro Mancosu

Italy

Kailash Manda

Japan

Giuliano Mariani

Italy

Patrizia Marini

Germany

James Marsh

United States of America

Gustavo Marta

Brazil

Jarad Martin

Australia

Maryanne Marymont

United States of America

Yukinori Matsuo

Japan

Martha Matuszak

United States of America

Charles Mayo

United States of America

Jean-Jacques Mazeron

France

Renaud Mazeron

France

Sean Mcbride

United States of America

Mark Mcdonald

United States of America

Alexander Mcewan

Canada

Helen Mcnair

United Kingdom

Philip Meijnen

Netherlands

Yusuf Menda

United States of America

Alejandra Mendez Romero

Netherlands

Martin Menten

Germany

Jens E. Meyer

Germany

Philipp T Meyer

Germany

Aisha Miah

United Kingdom

Gerald Mickisch

Germany

Matthias Miederer

Germany

Ben Mijnheer

Netherlands

George Mikhaeel

United Kingdom

Michael Milano

United States of America

Jeremy Millar

Australia

Michael Milosevic

Canada

Giuseppe Minniti

Italy

Rene-Olivier Mirimanoff

Switzerland

Darren Mitchell

United Kingdom

Koichi Miyake

Japan

Shin-Ichi Miyatake

Jordan

Paul Mobit

United States of America

Mohammad Mohammadianpanah

Iran

Valentina Monica

Italy

Michael Montemurro

Switzerland

Sabin Motwani

United States of America

Giannis Mountzios

France

Hossein Mozdarani

Iran

Karsten Muenstedt

Germany

John Mullen

United States of America

Anthony Mutsaers

Canada

Lee Myers

United States of America

Sten Myrehaug

Canada

Di Muzio Nadia

Italy

Motoo Nagane

Japan

Olaf Nairz

Austria

Takahito Nakajima

United States of America

Tatsuya Nakamura

Japan

Naoki Nakamura

Japan

Mustafa Naziroglu

Turkey

Mohamed Nazmy

Egypt

Karen Neelis

Netherlands

Ursula Nestle

Germany

John Ng

United States of America

Nam Nguyen

United States of America

Carsten Nieder

Norway

Thomas Nielsen

Denmark

Andrzej Niemierko

United States of America

Yuzuru Niibe

Japan

Jasper Nijkamp

Netherlands

Maximilian Niyazi

Germany

Takuma Nomiya

Japan

Remi Nout

Netherlands

Sandra Nuyts

Belgium

Joost Nuyttens

Netherlands

Hiroyuki Ogino

Japan

Etsuyo Ogo

Japan

Young-Taek Oh

Korea, South

Jiro Okami

Japan

Peter Olbert

Germany

Robert Olson

Canada

Chin Loon Ong

Netherlands

Hiroshi Onishi

Japan

Roberto Orecchia

Italy

Nicolas Orsi

United Kingdom

Oliver Ott

Germany

Natsuo Oya

Japan

Mahmut Ozsahin

Switzerland

Gokhan Ozyigit

Turkey

Roberto Pacelli

Italy

Tinsu Pan

United States of America

Renato G. Panizzon

Switzerland

Juan Pardo Montero

Spain

Matthew Parliament

Canada

Samir Patel

Canada

Baldev Patyal

United States of America

Arnold Paulino

United States of America

Ruth Paulssen

Norway

Timothy Peace

India

Huadong Pei

United States of America

Ans Pelgrims

Belgium

Jose Penagaricano

United States of America

Rodrigo Perez

Brazil

Richard Peschel

United States of America

Csilla Pesznyak

Hungary

Solange Peters

Switzerland

Heike Peulen

Netherlands

Steffi Ulrike Pigorsch

Germany

Michael Pinkawa

Germany

Paola Pinnarò

Italy

Marc D Piroth

Germany

Ludwig Plasswilm

Switzerland

George Plataniotis

United Kingdom

Thorsten Poeppel

Germany

Csaba Polgar

Hungary

Philip Poortmans

Netherlands

Bjoern Poppe

Germany

Richard Popple

United States of America

Edmond Pow

Hong Kong

Ramachandran Prabhakar

India

Simon Preuss

Germany

Hongryull Pyo

Korea, South

Bas Raaymakers

Netherlands

Dirk Rades

Germany

Eileen Rakovitch

Canada

Sara Ramella

Italy

Mark Ranck

United States of America

Coen Rasch

Netherlands

Abdul Rashid

United States of America

Elena Ratner

United States of America

Neal Ready

United States of America

Valerie Reed

United States of America

Sophie Renard-Oldrini

France

Zhong Renming

China

Truus Reynders

Belgium

Umberto Ricardi

Italy

Neil Richmond

United Kingdom

Anne Richter

Germany

Jolie Ringash

Canada

Sofia Rivera

France

Giovanna Rizzo

Italy

Mike Robbins

United States of America

David Roberge

Canada

Cliff Robinson

United States of America

Franz Rödel

Germany

Maria Rodriguez-Ruiz

Spain

Robert Rosenberg

Germany

Angeles Rovirosa

Spain

Volker Rudat

Saudi Arabia

Claudia Ruebe

Germany

Roswitha Runge

Germany

Jean-Claude Rwigema

United States of America

Samuel Ryu

United States of America

Nakamura Ryuji

Japan

Nabil Saba

United States of America

Haneen Sadick

Germany

Arjun Sahgal

Canada

Nami Saito

Germany

Shiro Saito

Japan

Elza Sakamoto-Hojo

Brazil

Joseph Salama

United States of America

Issam Saliba

Canada

Riccardo Santoni

Italy

Mitsuru Sasako

Japan

Otto Sauer

Germany

Cheng Saw

United States of America

Andrea Schaefer

Germany

Camilla Schalin-Jäntti

Finland

Alize Scheenstra

Netherlands

Cornelis Schilstra

Netherlands

Dominic Schinagl

Netherlands

Michel Schlienger

France

Rainer Schmid

Austria

Maximilian Schmid

Austria

Ralf Schneider

Switzerland

Reinhard Schulte

United States of America

Marco Schwarz

Italy

Roderich Schwarz

United States of America

David Schwwartz

United States of America

Marta Scorsetti

Italy

Therese Seierstad

Norway

Mehmet Sen

United Kingdom

Suresh Senan

Netherlands

Monika Serke

Germany

Thomas Seyfried

United States of America

Chirag Shah

United States of America

Masoud Shariat

Malaysia

Navesh Sharma

United States of America

Alan Shenkin

United Kingdom

Yuta Shibamoto

Japan

Jeng-Jer Shieh

Taiwan

Helen Shih

United States of America

Shinichi Shimizu

Japan

Igor Sirák

Czech Republic

Ben Slotman

Netherlands

Deedee Smart

United States of America

Robert Jan Smeenk

Netherlands

Winnie So

China

Matthias Soehn

Germany

Antje Sommerer

Germany

Lena Specht

Denmark

Paul Sperduto

United States of America

Jacco Steenhuijsen

Netherlands

Ulrich Steger

Germany

Robin Stern

United States of America

Florian Sterzing

Germany

Florian Stieler

Germany

Guy Storme

Belgium

Heidi Stranzl

Austria

Jonathan Strauss

United States of America

Lidia Strigari

Italy

Herwig Strik

Germany

Vratislav Strnad

Germany

Silvia Stuart

Brazil

Gabriela Studer

Switzerland

Martin Stuschke

Germany

Changqing Su

China

Bogdana Suchorska

Germany

Yelin Suh

United States of America

Stephane Supiot

France

Jamema Swamidas

India

Reinhart Sweeney

Germany

Henry Sze

Hong Kong

Alphonse Taghian

United States of America

Patricia Tai

Canada

Diana Tait

United Kingdom

Izumi Takeyoshi

Japan

Colin Tang

Australia

Tawee Tanvetyanon

United States of America

Martin Taphoorn

Netherlands

Juan Tardío

Spain

Mojgan Taremi

Canada

Carolyn Taylor

United Kingdom

Mikko Tenhunen

Finland

Chris Terhaard

Netherlands

David Terrian

United States of America

Jeremy Tey

Singapore

Kiki Theodorou

Greece

CR THOMAS

United States of America

Jakob Thomsen

Denmark

Frits Thorsen

Norway

Daniela Thorwarth

Germany

Beate Timmermann

Germany

José-Luis Tisaire

Spain

Ranjini Tolakanahalli

United States of America

Natsuo Tomita

Japan

Emily Tonorezos

United States of America

Erkan Topkan

Turkey

Mahmoud Toulany

Germany

Ryo Toya

Japan

Charlene Treanor

United Kingdom

Almut Troeller

Germany

Scott Tyldesley

Canada

Katherine Tzou

United States of America

Yee Ung

Canada

Keith Unger

United States of America

James Urbanic

United States of America

Jitsuo Usuda

Japan

Maurizio Valeriani

Italy

Rick Van De Langenberg

Netherlands

Uulke Van Der Heide

Netherlands

Hans Paul Van Der Laan

Netherlands

Yvonne Van Herten

Netherlands

Emile Van Lin

Netherlands

Judith Van Loon

Netherlands

Peter Van Luijk

Netherlands

Aart Van T Veld

Netherlands

Stanislav Vatnitskiy

Austria

Peter Vavassis

Canada

Hansjoerg Vees

Switzerland

Liv Veldeman

Belgium

Michael Velec

Canada

Vaneja Velenik

Slovenia

Michael Veness

Australia

Wilko Verbakel

Netherlands

Lia Verhoef

Netherlands

Joost J.C. Verhoeff

Netherlands

Bhadrasain VIKRAM

United States of America

Asgaut Viste

Norway

Peter Voet

Netherlands

Michael Vogelbaum

United States of America

Ivan Vogelius

Denmark

Dirk Vordermark

Germany

Jacqueline Vuky

United States of America

Phyllis Wachsberger

United States of America

Hitoshi Wada

Japan

Margaret Wallington

Australia

Xiaochun Wang

United States of America

Dongxu Wang

United States of America

Ute Warnecke-Eberz

Germany

David Waterhouse

Australia

Damien Charles Weber

Switzerland

George Weiner

United States of America

Christian Weiss

Germany

Grit Welzel

Germany

Zhifei Wen

United States of America

Thomas G. Wendt

Germany

Frederik Wenz

Germany

Lamberto Widesott

Italy

Jürgen Wilbert

Germany

Frank Willeke

Germany

Jacqueline Williams

United States of America

Elena Wilson

United Kingdom

Sebastian Winklhofer

Switzerland

Jeffrey Wong

United States of America

Binbin Wu

United States of America

Q. Jackie Wu

United States of America

Vincent Wu

Hong Kong

Ching-Fang Wu

Taiwan

Peter Wust

Germany

Nikolaos Xenidis

Greece

Shokei Yamada

United States of America

Tetsuya Yamamoto

Japan

Hideya Yamazaki

Japan

Deshan Yang

United States of America

Catheryn Yashar

United States of America

Linda Yasui

United States of America

Richard` Yeo

Singapore

Seung-Gu Yeo

Korea, South

Sun Yi

United States of America

Ferah Yildiz

Turkey

Torunn Yock

United States of America

Sua Yoo

United States of America

Ryoichi Yoshimura

Japan

Xue Qin Yu

Australia

Ning Yue

United States of America

Joanna Zawacka-Pankau

Poland

Sadamoto Zenda

Japan

Zhao-Chong Zeng

China

Tiezhi Zhang

United States of America

Jian Dong Zhao

China

Tingliang Zhuang

United States of America

Thomas Zilli

Switzerland

Jennifer Zook

United States of America

Eduardo H. Zubizarreta

Austria

